# Applications of Single Atom Catalysts for Environmental Management

**DOI:** 10.3390/ijerph191811155

**Published:** 2022-09-06

**Authors:** Rongkui Su, Hongguo Zhang, Feng Chen, Zhenxing Wang, Lei Huang

**Affiliations:** 1 College of Environmental Science and Engineering, Central South University of Forestry & Technology, Changsha 410004, China; 2 Power China Zhongnan Engineering Corporation Limited, Changsha 410004, China; 3 School of Environmental Science and Engineering, Guangzhou University, Guangzhou 510006, China; 4 School of Environmental and Biological Engineering, Henan University of Engineering, Zhengzhou 451191, China; 5 South China Institute of Environmental Sciences, Ministry of Ecology and Environment of the People’s Republic of China, Guangzhou 510655, China

With the rapid development of industrialization, human beings have caused many negative effects on the environment that have endangered the survival and development of human beings, such as the greenhouse effect, water pollution, energy depletion, etc. [1,2,3]. It is imperative to seek scientific means to control them. Nanotechnology has driven scientific advances in catalytic materials and processes over the years, and the unique physicochemical and electronic properties of materials ranging from bulk to nanoscale have led to a wide range of applications, including environmental remediation [4]. The single-atom catalyst (SACs) is a new frontier material for environmental remediation applications, other than nanomaterials [5]. Since the successful synthesis of single-atom Pt catalysts (Pt_1_/FeO_X_) by Zhang and coworkers in 2011 [6], which started the “gold rush” of research on SACs by domestic and foreign teams, and developed SACs with the progress of research, SACs have been applied in many fields such as biomedicine, environmental protection, and energy conversion [7,8,9].

Homogeneous catalysis has the characteristics of 100% utilization of catalytic atoms, high homogeneity, high selectivity, and high activity, but there are some problems such as difficult recovery and poor stability [10]. Heterogeneous catalysts have the advantages of easy recovery and good stability, but there are some problems such as low utilization of catalytic atoms, poor activity, and poor selectivity [4]. SACs have nearly 100% atom utilization and easy separation properties, which can build a bridge between homogeneous catalysis and heterogeneous catalysis, combining the advantages of both [11]. In addition, SACs can also exhibit unique catalytic properties in some reactions, such as excellent activity, selectivity, and stability [12,13,14,15]. An excellent catalyst needs the ability to adapt to various complex conditions, and excellent stability is the basis of efficient catalysis, while SACs show good resistance to high temperature, acid and alkali, and organic solvents [16,17,18,19,20]. Catalytic selectivity is the key to many industrial production processes, but it is often difficult to control, and SACs have exactly this feature [21,22,23]. Single-atom catalysts can reduce the number of metals used for surface reactions and often exhibit significant selectivity, which is not exhibited by the corresponding nanoparticles [24,25]. Single-atom catalysts have both the advantages of easy separation and isolated active sites similar to homogeneous catalysts, resulting in high catalytic activity [26,27,28,29,30]. However, the catalytic activities of different metal single-atom catalysts are not consistent. Due to their ultra-high performance, environmental friendliness, structural/chemical stability, and maximum utilization of active metal centers, SACs have become an extremely important material in the field of environmental catalysis [31].

The ultra-high surface energy of a single atom requires the support and interaction of the carrier to maintain monodispersity [32]. The specific surface area of the SACs carrier, the content of single-atom immobilization sites, the interaction force between the immobilization sites and the metal atoms, and the properties of the carrier itself are the keys to determining the loading, stability, and catalytic performance of SACs. And the SACs of different carriers usually exhibit different catalytic properties. Therefore, the carrier plays a crucial role in various properties of SACs. At present, the commonly used single-atom catalyst carriers include metal carriers [6,20,33,34,35], metal compound carriers [36,37,38,39,40], and non-metal carriers [41,42,43,44,45].

Today, teams from all over the world have developed a series of methods for preparing single-atom catalysts, such as co-precipitation, impregnation, and chemical vapor deposition [46,47,48,49,50,51,52]. Compared with traditional supported catalyst preparation methods, the preparation of single-atom catalysts should not only ensure the successful loading of the active components of the catalyst but also control the additional amount of the active components of the catalyst to ensure that the prepared catalyst reaches the atomic level [53,54]. The arc-discharge method is to initiate arc discharge by moving the anode to the cathode, and the anode evaporates at the high temperature generated by the arc so that charged particles are deposited on the surface of the cathode, thereby preparing the desired catalyst [55]. Strong electrostatic adsorption is a technique that has been used to create highly dispersed metal nanoparticles on conventional supports, relying on electrostatic interactions with the carrier surface to selectively deposit metal precursors [56]. Flame spray pyrolysis is a new method for preparing single-atom catalysts, and it is a technique that can effectively synthesize metal nanoparticles of uniform size. The preparation process is to mix the precursors in the solution, which enables all the precursors to be uniformly mixed at the atomic level in the initial phase, and the metal salt solution is sprayed into the high-temperature flame generated by the combustion of the fuel gas in the form of water mist. Two or more substances are then loaded together to produce a catalyst [57]. Atomic layer deposition, with its enormous ability to precisely control the deposition of single atoms and nanoclusters, is a powerful method to study the relationship between catalyst structure and catalytic performance—better activity, selectivity, and longevity [58].

The synthesis mechanism of single-atom catalysts has always been one of the focuses of development. Due to the size effect of single atoms, the corresponding surface energy is relatively high, and isolated single atoms can easily migrate and aggregate into particles. Therefore, to achieve single-atom dispersion, it is not only necessary to choose a suitable carrier to interact with the metal, but also an appropriate anchoring strategy to better anchor the metal atoms to avoid aggregation [59]. Therefore, exploring the anchoring strategy of single-atom catalysts is also very important for the synthesis of SACs. In general, Zhao et al. [60] classified anchor sites into five types: doped heteroatoms, defect sites, surface atoms, cavity sites, and metal sites.

Research on SACs for environmental remediation is still at an early stage and faces great opportunities and challenges [5]. Although the potential catalytic mechanisms have some similarities, the successful transformation of SACs in other fields change into environmental applications such as polluted air and water treatment is largely unknown. The current literature clearly shows that the improvement of catalytic activity and selectivity depends on the type of target reaction and its mechanism. The characteristics of SACs (such as the coordination environment of metal atoms, the adsorption interaction with reactants and intermediates, and the charge interaction between metals and carriers) determine their performance in environmental applications. By matching the actual needs of environmental remediation and the advantages of sacs based on the above-mentioned basic catalytic mechanism, potential environmental applications include Fenton-like reaction [55,61,62], electrocatalytic water treatment [56,63,64], dehalogenation [57,65], nitride reduction [66,67], photocatalytic treatment [58,68,69], removal of oxygen-containing acid ions [70,71], degradation of volatile organic compounds (VOCs) [72,73], etc.

Although SACs have shown good potential in environmental treatment, there are still many key problems to be solved before realizing the wide application of monatomic catalysts. (1) Cost of equipment and materials for preparing SACs is too high to meet the requirements of industrial production. (2) Stability and activity of SACs in local environments need to be verified for a long time. (3) SACs are faced with low metal loading and poor single atom homogeneity, which limit the development of SACs. (4) Catalytic mechanism of SACs is not clear enough, the regulation law of single atoms on active species is not clear, and the synergistic effect between double atoms and multi atoms is unclear.

This Special Issue is built to promote the researchers who deliver these thoughts, ideas, and discoveries of SACs in environment applications. Thank you to everyone who wants to, or can contribute to this Special Issue.
